# Antimicrobial Prescription Behavior in Equine Asthma Cases: An International Survey

**DOI:** 10.3390/ani14030457

**Published:** 2024-01-30

**Authors:** Astrid J. van den Brom-Spierenburg, Alexandra N. Mureșan, Cornélie M. Westermann

**Affiliations:** 1 Department of Clinical Sciences, Faculty of Veterinary Medicine, Utrecht University, Yalelaan 114, 3584 CM Utrecht, The Netherlands; 2 Department of Internal Medicine, Faculty of Veterinary Medicine, University of Agricultural Sciences and Veterinary Medicine of Cluj-Napoca, 400372 Cluj-Napoca, Romania; alexandra.muresan@usamvcluj.ro

**Keywords:** antibiotic, horse, respiratory disease, antimicrobial stewardship, inflammatory airway disease, recurrent airway obstruction

## Abstract

**Simple Summary:**

It is important to minimize and optimize the use of antimicrobials to prevent resistance to these medications. Resistant bacteria can cause dangerous infections in people or animals. As in people, asthma in horses is considered non-infectious, so it is not necessary to use antimicrobials. However, in this this large survey completed by 249 equine veterinarians in 25 countries, we found that, just like general practitioners sometimes prescribe antimicrobials in human cases, 53% of the responding veterinarians stated to sometimes prescribe antimicrobials in asthma cases. We also tried to find out why and it became clear that knowledge of the pathophysiology of asthma, the country/culture the veterinarian works in, and the type of practice that they work in are important factors influencing prescription behavior. On the patient level, medical factors clearly predominate, possibly because several of these could also be compatible with infectious disease. This study underlines the need for further research on antimicrobials in equine asthma, the dissemination of knowledge of the pathophysiology of equine asthma, and clear (worldwide) guidelines on antimicrobial stewardship to diminish the inappropriate use of (critically important) antimicrobials in cases of equine asthma.

**Abstract:**

Equine asthma is considered a non-infectious respiratory disease characterized by inflammation and hypersensitivity. Given the importance of antimicrobial stewardship, an international survey was designed to investigate the use of antimicrobials in asthmatic horses and the factors influencing prescription behavior. An online survey was distributed in six languages by international mailing lists and social media from December 2020 to January 2022. Of the 434 responses recorded, 249 veterinarians working in 25 countries finished the survey. These included 79 ECEIM/ACVIM diplomats. A total of 204 respondents confirmed national regulations concerning antimicrobial use in their country. Knowledge of the asthma definitions as presented in the revised ACVIM consensus statement was greater in veterinarians treating over 95% of equine patients compared to veterinarians treating more species, based on 10 questions (answers consistent with the consensus statement in 7 (IQR 5–9) and 4 (IQR 3–6) questions, respectively, (*p* < 0.001)). A total of 131 respondents stated to use antimicrobials (at least ‘sometimes’) in at least one of the three presented cases consistent with equine asthma. Trimethoprim-Sulfa combinations, penicillin(s), and tetracyclines were prescribed most (by 105, 53 and 38 veterinarians, respectively). Aminoglycosides, cephalosporins and fluoroquinolones were also selected (22, 15, 9 veterinarians respectively). Tetracyclines and cephalosporins are prescribed more often by veterinarians working without national regulations (*p* 0.019 and *p* 0.035, respectively). The most selected factors influencing prescription behavior were: ‘tracheal wash culture’ (62% of 131 veterinarians using antimicrobials in these cases), ‘whether other horses in the barn have similar complaints’ (60%), and ‘response to other forms of therapy’ (53%). In conclusion, insight into prescription behavior is the first step towards minimizing and optimizing antimicrobial use.

## 1. Introduction

Asthma is a complex and heterogeneous disorder. Both equine and human asthma are characterized by chronic airway inflammation, bronchospasm and similar asthma-associated airway remodeling, with comparable causes of sensitization and exacerbation. Therefore, Equine Asthma (EA) is a unique animal model for human asthma [1,2].

In general, antimicrobials are not expected to be effective in the context of most human asthma exacerbations, and their routine use is not recommended [3,4]. This is supported by a Cochrane review (2018), in which insufficient evidence was found to support antimicrobial use in human asthma [5].

In specific subsets of asthmatic children, macrolides show some positive effects in prevention and treatment, maybe due to their anti-inflammatory properties [6,7,8]. Some positive effects of antimicrobials are also reported in adults; in a large human retrospective cohort study in an adult hospitalized population, the combination of antimicrobials and oral corticosteroids (CS) was associated with a lower risk of treatment failure compared to matched patients treated with only oral CS [9].

Despite the limited and conflicting evidence, guidelines discouraging antimicrobial use, and the knowledge that only a small percentage of acute asthma cases is associated with evidence of bacterial infection, it appears that 32% of children and over 40% of adult asthma patients are treated with antimicrobials as well as CS [3,9,10].

In horses, a small study in patients with severe equine asthma showed that the macrolide azithromycin decreased the expression of IL-8 and IL-1β but failed to improve the other variables evaluated [11]. The same antimicrobial also reduced airway neutrophilia but did not improve lung function or reduce smooth muscle mass in the central lung [12]. Another small study using ceftiofur in horses with severe equine asthma and a positive tracheal wash culture showed some improvement in the clinical score and MPO activity, but there was no effect on other parameters [13].

The importance of antimicrobial stewardship (i.e., the judicious use of antimicrobials) is emphasized by the estimated annual 2.8 million antimicrobial-resistant infections in the USA in human beings [14]. Suboptimal or unnecessary use of antimicrobials, which is estimated to be the case in 30% of all antimicrobials prescribed in hospitals, contributes to the development of these infections [15].

In veterinary medicine, antimicrobial use should address the clinical needs of patients with clear signs, symptoms, or laboratory test results indicating a bacterial infection. The type of antimicrobial should be carefully selected; prescribers should prioritize the use of antimicrobials with the lowest importance to human medicine (published as the ‘Critically Important Antimicrobials for Human Medicine’ list) to preserve the effectiveness of important antimicrobials for both veterinary and human medicine [16,17].

The World Health Organization (WHO) recognizes that recommendations may vary from country to country and that national documents may overrule WHO guidelines. Apart from national regulations and guidelines, antimicrobial prescribing guidelines for equine syndromes can be very valuable and are becoming increasingly common. These present the most up-to-date information on appropriate therapy [18,19,20].

Unfortunately, inappropriate use (regarding indication, dosing, and/or type) of antimicrobials is also common in equine practice. For example, the administration of antimicrobials to horses with signs of asthma (cough and nasal discharge) without clinical signs suggesting infection was reported [21,22]. While overall antimicrobial usage seemed to reduce in the past years and protocols seemed to be introduced more, the practice of prescribing critically important antimicrobials is still widespread, according to a recent study by Wilson et al. (2023) [23].

An increasing number of studies is being published on prescription behavior in both human and veterinary medicine [23,24]. Concerning human asthma, examples of patient-related factors determining prescription behavior are lung auscultation, unusual findings, uncertainty regarding the diagnosis, fear of deterioration, age of the patient, and not having biomarker testing available. Non-patient-related factors are, for example, fear of litigation, awareness of and agreement with existing guidelines, and career length [25,26,27]. Although aware of the risk of antimicrobial resistance, physicians rarely seemed to take that into account when considering antimicrobials [27].

A combination of factors may drive equine veterinarians to prescribe antimicrobials for equine asthma. In the literature, several factors are reported. Examples are (perceived) pressure from clients, fear of reduced performance or even deterioration in a patient leading to litigation, the idea that antimicrobials are unlikely to cause harm, insufficient knowledge of guidelines, and the suggestion that airway inflammation is commonly caused by bacterial infection, particularly in young (yearling and two-year-old) racehorses [28,29,30].

This study has a fourfold aim. A survey for equine veterinarians was developed to:

− gain more insight into the prescription rate for antimicrobials in Equine Asthma worldwide;
− determine which characteristics of the veterinarians are associated with antimicrobial (AM) use; for instance, the effect of the country, career length, or national guidelines;
− determine the effect of medical knowledge on the decision-making;
− identify the case-related (medical and non-medical) factors that drive equine veterinarians towards or against the prescription of antimicrobials.

We hypothesize that education level and awareness of published protocols have significant consequences on prescription behavior in the participants and that both medical and non-medical factors play a role.

## 2. Materials and Methods

A survey was designed with Qualtrics software “Manufacturer Qualtrics XM (Amsterdam, The Netherlands, 2020–2022)” (see Supplementary Materials for the complete survey), consisting of several parts:

Part 1 contained an informed consent form and questions concerning veterinarian characteristics like career length, percentage of equine patients, education, type of practice, diagnostic modalities used, and country. It was included whether or not their country and/or practice had (national) regulations concerning antimicrobial use and how they were influenced by them (guided/limited).

In Part 2, ten questions were asked regarding the definition and pathophysiology of equine asthma as presented in the last revised consensus statement [31]. For all questions, there were five answer options: the correct answer, three distractors, and ‘I do not know’. For nine questions, there was one true right answer, but with new literature being published (2022) concerning remodeling of airway smooth muscle in the equine airways in mild to moderate equine asthma (EA), the authors decided that in question nine, ‘Airway remodeling is a factor in…’ not only the answer ‘severe equine asthma’ was considered correct, but also the answer ‘both mild to moderate and severe equine asthma’ [32]. In this part, a question was also included concerning the respondent’s opinion on the role of different pathogen types in the etiology of equine asthma.

Part 3a consisted of three cases representing three forms of equine asthma (severe, mild, and moderate, respectively) as based on the consensus statement [31]; see text Box 1, Box 2 and Box 3. Respondents were asked to define the cases. Options were asthma (including a severity/type) and other terms like ‘recurrent airway obstruction (RAO)’ or ‘inflammatory airway disease (IAD),’ in which selection of multiple answers was possible. Participants were also asked whether they would use antimicrobials in each case, with a 5-point-verbal rating scale (VRS) from 0 (never), 1 (sometimes), over 2 (half of the time), to 3 (most of the time), and 4 (always). If so, they were asked which antimicrobials they would use. Respondents who would at least ‘sometimes’ use antimicrobials in at least one of the cases went through to Part 3b.

Box 1 **Case 1: A case representing Severe Equine Asthma.**

A 13 yo Dutch Warmblood gelding, used for pleasure riding, is presented to you because he has been coughing for the last two weeks. Last year, in the same period, he had similar complaints that improved on your advice and treatment. He is housed on pasture during the day and stabled at night. The horse is bright and alert, has a good body condition, a rectal temperature of 37.8 °C (100 °F), some nasal discharge (white mucus), and shows increased breathing effort (frequency 22/min, abdominal). He has a frequent, productive cough. On auscultation, you hear increased breath sounds throughout the thorax and mild rhonchi on the trachea and lungs.

Airway endoscopy reveals marked amounts of mucus (grade 4/5).

The BAL result is: Macrophages 43%, Lymphocytes 29%, Neutrophils 27%, Mast cells 1%, Eosinophils 0%.

Box 2 **Case 2: A case representing Mild Equine Asthma.**

A 3 yo thoroughbred mare is presented to you for recent suboptimal performance (racing). She has occasionally coughed for a week now. A few other horses in the barn have been heard coughing as well. She is housed in a stable on wood shavings and eats good-quality hay. She does not have a fever (37.6 °C/99.7 °F). You did not find any orthopedic or cardiovascular abnormalities. On examination of the respiratory tract, you find a normal breathing frequency (12/min), no dyspnea, and no adventitious sounds on auscultation of the thorax and trachea. A little bit of seromucous nasal discharge is present in both nostrils.

On endoscopy, you see pharyngeal lymphoid hyperplasia (grade 3: pink and white follicles covering the pharyngeal walls and dorsal surface of the soft palate) and a mild increase in mucus in the trachea (grade 2/5).

The BAL result is: Macrophages 57%, Lymphocytes 33%, Neutrophils 4%, Mast cells 2%, Eosinophils 4%.

Box 3 **Case 3: A case representing Moderate Equine Asthma.**

A 9 yo German Warmblood mare, a high-level show-jumper, is presented to you because of poor performance. Especially at the last fences, she seems to have more touches and faults. Also, the owner noticed that it takes her longer to regain a normal breathing frequency after exercise. Coughing has been heard mainly at the beginning of exercise. All complaints have been present since approximately 2 weeks. At rest, the general and respiratory clinical exams are unremarkable.

On endoscopy, you see increased mucus production (grade 3/5).

The BAL result is: Macrophages 49%, Lymphocytes 28%, Neutrophils 16%, Mast cells 6%, Eosinophils 1%.

In Part 3b, they were asked to select the factors that would influence their decision to use antimicrobials or not. For all the factors they selected, they were asked how this factor would influence their decision: a 5-point-VRS from −2 (strongly against) to −1 (against) over 0 (neutral) to 1 (in favor) and 2 (strongly in favor) of antimicrobial use. For some factors, the findings were divided in order to obtain more insight; for example, if the factor ‘auscultation’ was selected, the respondent was asked how ‘normal breath sounds on auscultation’ would influence the decision, but also, e.g., ‘rhonchi on auscultation’. Thirty factors were presented, designed by authors AS and CW and partly based on a similar human study [26]. These included clinical factors (e.g., coughing) and non-clinical factors (e.g., whether or not the horse is insured). Factors guiding toward a diagnosis other than asthma (e.g., fever) were not included. Of two factors (‘owner request for antimicrobials’ and ‘it is general practice in our clinic to prescribe antimicrobials in these cases’), participants were, if they did not select that factor, asked to select why not; do they not encounter this factor or are they not influenced by it?

In Part 4 (visible to all respondents), the survey ended with the possibility of leaving an e-mail address for future updates on the results and/or collaboration in future research.

The survey was designed in English and translated by veterinarians who were native speakers of Dutch (AS), French (AT), Italian (SB), Spanish (MM), and Romanian (ANM).

The link to the survey was distributed by e-mail through several national professional bodies and by the ACVIM listserve, an electronic mailing list for ACVIM (American College of Veterinary Internal Medicine) and ECEIM (European College of Equine Internal Medicine) Diplomats, and on social media (Facebook^®^).

Results were exported to Excel^®^ version 16.0 (Manufacturer Microsoft, Redmond, WA, USA) and anonymously analyzed using the statistical software package SPSS 27.0 (Manufacturer IBM Corp., Armonk, NY, USA). Univariate analyses were performed by exploring associations (Chi-square, Fisher’s exact) and groupwise and pairwise comparisons for ordinal data (Mann–Whitney U, Kruskal–Wallis). To display the different responses across geographical areas, a choropleth map was created using Datawrapper free online software by Datawrapper GMBH (version 29-1-2024 Berlin, Germany) and then edited with Microsoft PowerPoint^®^ 2019 (Manufacturer Microsoft, Redmond, WA, USA). Both Excel^®^ and PowerPoint^®^ were also used for the figures and tables.

## 3. Results

### 3.1. Respondent Characteristics

#### 3.1.1. Personal, Career and Working Environment

**Figure 1:**
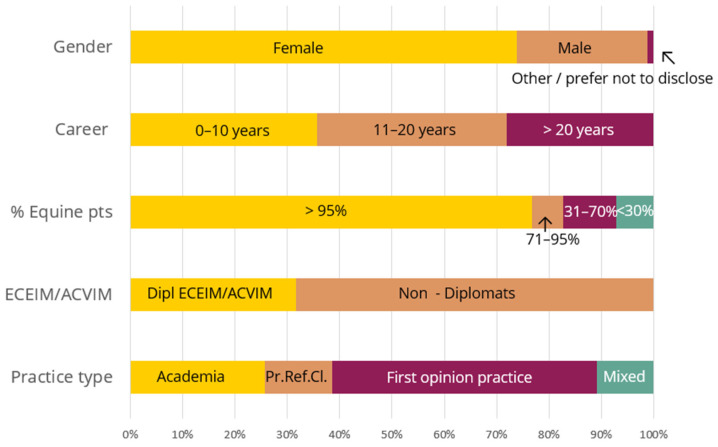
Respondent characteristics (*n* = 249). Pts stands for patients, Pr.Ref.Cl. for private referral clinic.

Of the 434 responses recorded (from December 2020 to January 2022), 249 were complete (57%). The data from these 249 responses were analyzed. Seventy-four percent of the respondents (*n* = 184) identified as female, 24% (*n* = 62) as male, and 1% (*n* = 3) as ‘other/prefer not to disclose’. The career length was equally divided amongst respondents: 36% (*n* = 89) practiced for 0–10 years, 36% (*n* = 90) for 11–20 years, and 28% (*n* = 70) for over 20 years. The largest proportion (77%, *n* = 191) were equine veterinarians (with >95% of their patients being equids); the other groups were smaller (<30% equids 7%, *n* = 18; 31–70% equids 10%, *n* = 25, 71–95% equids 6%, *n* = 15). Seventy-nine respondents (32%) were diplomats of the American College of Veterinary Internal Medicine (ACVIM) or European College of Equine Internal Medicine (ECEIM). Most respondents worked in first opinion practice (FOP) (126; 51%), 64 (26%) worked in academia, 32 (13%) in a private referral clinic (PRC), and the remainder (27; 11%) in a combination of these practice types (see Figure 1).

#### 3.1.2. Diagnostic Modalities

Of the ancillary diagnostic modalities, endoscopy was selected most (206 respondents, 83%), followed by bronchoalveolar lavage (BAL) (*n* = 190, 76%), ultrasound (*n* = 148, 59%), tracheal wash (*n* = 136, 55%) and radiology (*n* = 99, 40%). Proportions of veterinarians using these techniques in different practice types (FOP, PRC and Academia) were similar to the proportion of respondents not working (solely) in that practice type, except for the tracheal wash; this was performed less often by veterinarians in FOP (*n* = 61, 48%) compared to veterinarians in other practice types (*n* = 75, 61%, *p* 0.047), while respondents working in private referral clinics perform tracheal washes more often in our cases (23/32; 72%) compared to veterinarians in other practice types (113/217; 52%, *p* 0.036).

Additional pressure measurements and/or pulmonary function tests (*n* = 18) were used equally in different practice types. With the option ‘other’ workup modalities, 25 respondents report bloodwork: 13 unspecified or specified as hematology or clinical chemistry (inflammatory proteins, SAA), 11 specified as blood gas analysis, and one specified as measuring lactate.

#### 3.1.3. Geographical Distribution

**Figure 2:**
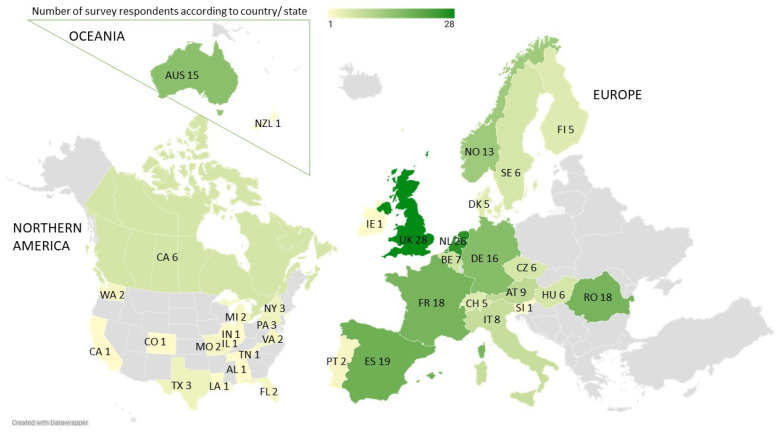
Geographical distribution of respondents over the world. Not pictured Uruguay (n = 1), South Africa (n = 1). Abbreviations were chosen according to country codes: Australia AUS, Austria AT, Belgium BE, Canada CA, Czechia CZ, Denmark DK, Finland FI, France FR, Germany DE, Hungary HU, Ireland IE, Italy IT, The Netherlands NL, New Zealand NZL, Norway NO, Portugal PT, Romania RO, Slovenia SI, Spain ES, Sweden SE, Switzerland CH, United Kingdom UK. US States were abbreviated also according to their country codes: Alabama AL, California CA, Colorado CO, Florida FL, Illinois IL, Indiana IN, Louisiana LA, Michigan MI, Missouri MO, New York NY, Pennsylvania PA, Tennessee TN, Texas TX, Virginia VA, Washington WA.

The respondents were working in 25 different countries, of which 199 participants (80%) were in 19 countries in Europe and 26 (10%) of them in 15 states in the USA (Figure 2).

#### 3.1.4. National Regulations and Practice Guidelines

Awareness of national regulations concerning antimicrobial use in their country was stated by 204 (82%) respondents from 24 countries.

Practice regulations are present according to 115 (46%) of respondents; no significant differences were found between the practice types these respondents were in.

Most participants who stated the presence of national regulations or practice guidelines take these into account in every case in which they consider antimicrobials (170/204, 83% and 97/115, 84%, respectively). The majority feels guided by the national regulations (159/204, 78%) and the practice guidelines (94/115, 82%), but some of the participants feel limited by these national regulations (74/204, 36%) and practice guidelines (26/115, 23%).

### 3.2. Questions Regarding the Pathophysiology of Asthma

#### 3.2.1. Questions Regarding the ‘Equine Asthma Consensus Statement’

Over all participants, the median number of correct answers was 6/10 (interquartile range, IQR 4–9). The number of correct answers was higher in the group of ACVIM/ECEIM diplomats (9, IQR 7–10) compared to non-diplomats (5, IQR 4–7, *p* < 0.001). In academia, respondents scored higher (9, IQR 8–10) compared to colleagues in FOP (4, IQR 4–6, *p* < 0.001). Also, respondents in a private referral clinic scored higher (8, IQR 7–9) compared to participants working in FOP (*p* < 0.001).

No differences were found between groups with different career lengths.

Equine veterinarians (>95% equine patients) had a higher median score (7, IQR 5–9) compared to both the group with <30% equine patients (4, IQR 3–4, *p* < 0.001) and the group with 30–70% equine patients (5, IQR 3–6, *p* 0.01), and when compared to the group with 71–95% equine patients (4/10 correct answers (IQR 4–6.5)), a similar trend was present (*p* 0.054).

#### 3.2.2. Respondents’ Opinions on the Role of Pathogens in the Pathophysiology of Equine Asthma

**Figure 3:**
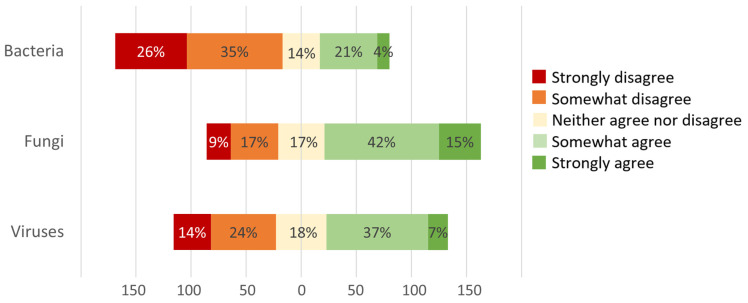
The answers of respondents to the statement: “I assume an important role for [bacteria/fungi/viruses] in some Equine Asthma cases”. Total *n* = 249.

Fifty-seven percent of respondents somewhat or strongly agreed with the statement, “I assume an important role for fungi in some equine asthma cases”. Forty-four percent and 25% agreed on the same statement concerning viruses and bacteria, respectively. See Figure 3.

### 3.3. Cases

#### 3.3.1. Case Definition and Antimicrobial Use

##### Case Definition

In case 1, 154 (62%) of the respondents chose one definition, as did 146 (59%) in case 2, and 153 (61%) in case 3. The remaining respondents chose more than one definition (range of 0–6 options per case per respondent). The responses are presented in Table 1.

Only 118 (47%) defined all three cases as asthma, in line with the consensus statement [31]. Two hundred and twenty-six respondents (91%) defined at least one of the cases as asthma. No difference was found between Europe, the USA/Canada, and Australia/New Zealand with regard to the use of the definition ‘Equine Asthma’ nor between different parts of Europe.

In cases 1 and 3, representing severe and moderate equine asthma, respectively, 76% (*n* = 189) and 77% (*n* = 191) of the respondents considered the cases ‘equine asthma’. In case 2, representing mild equine asthma, only 58% (*n* = 144) considered the patient to be suffering from Equine Asthma. When taking type/severity of asthma into account, in case 1, 94 (38%) of respondents chose ‘Severe Equine Asthma (SEA)’ in line with the consensus statement. Thirty-nine percent of all participants (*n* = 96) defined case 2 as ‘mild equine asthma’, in line with the consensus statement. Sixty percent of participants (*n* = 149) chose ‘Mild Equine Asthma’ and/or ‘Mild to Moderate Equine Asthma’ when asked to define case 3.

As shown in Table 1, the previously advised terms (former version consensus statement (2007) [33]) for equine asthma: Inflammatory Airway Disease (IAD) and Recurrent Airway Obstruction (RAO) are still used by many veterinarians. One hundred and twenty-six respondents (51%) used the terms RAO and/or SEA to define case 1, 169 (68%) used IAD and/or Mild EA to define case 2, and 189 (76%) used IAD and/or Mild to moderate EA and/or Moderate EA to define case 3.

People who used the asthma definition (*n* = 226) gave more correct answers to the knowledge questions (median 6, IQR 4–9) than people who did not consider any of the cases asthma (*n* = 23, median 4, IQR 4–5.5, MWU, *p* 0.002). People who defined all three cases as asthma gave more correct answers (median 8 (6–9), *n* = 118) than people who defined none (median 4 (4–5.5), *n* = 23, *p* < 0.001), one (median 4 (3–5), *n* = 46, *p* < 0.001), or two cases as asthma (median 5 (4–7), *n* = 62, *p* < 0.001).

##### Antimicrobial Use

Overall, 131 respondents (53%) use antimicrobials at least ‘sometimes’ in at least one of the cases. Sixty-three (25%) only in one case, 28 (11%) in two of the cases, and 40 respondents stated to use antimicrobials (16%) in all three cases (at least sometimes).

**Figure 4:**
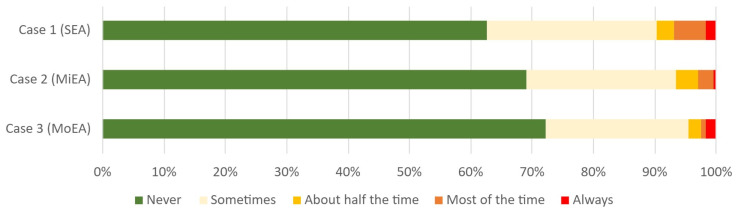
The answers to the question: “Do you treat this horse (or similar cases) with antimicrobials?” in the three presented cases. *n* = 249.

The distribution of the responses to the question: “Do you treat this horse (or similar cases) with antimicrobials?” with the options ‘never’, ‘sometimes’, ‘about half the time’, ‘most of the time’ and ‘always’ per case are shown in Figure 4. The proportion of respondents that stated to never use antimicrobials is largest (72%, *n* = 180) in case 3 (MoEA), smaller (69% *n* = 172) in case 2 (MiEA), and the smallest in case 1 (SEA) with 63% (*n* = 156) all *p* < 0.001).

Antimicrobial use, defined as stating to use antimicrobials at least sometimes in at least one of the cases, was compared between groups of respondents based on several respondent characteristics.

The number of questions on the pathophysiology of asthma that were correctly answered was lower in the participants using antimicrobials (5, IQR 4–8) compared to participants never using antimicrobials (7, IQR 5–9).

No difference was found between veterinarians with different career lengths or between participants who were or were not aware of national regulations concerning antimicrobial use in their countries (Table 2). There was a trend towards less use by ECEIM/ACVIM diplomats (Table 2).

Of the participants who considered bacteria important in the pathophysiology of (at least part of the) asthma cases, a larger proportion (81%, *n* = 51) stated to use antimicrobials than of the people who did not think bacteria were a causal factor (43%, *n* = 80). In academia, fewer veterinarians stated to use antimicrobials (41%, *n* = 29) vs. 57% (*n* = 102) of people working outside academia. In first-opinion practice (FOP), more veterinarians use antimicrobials (63%, *n* = 79) in these cases than people not working in FOP (42% *n* = 52). The proportion of equine veterinarians (of which >95% of their patients are equine) using antimicrobials (47%, *n* = 90) is smaller when compared to colleagues who treat several species (71%, *n* = 41). People using the definition ‘asthma’ for the cases used fewer antimicrobials; for the group as a whole, that was not significant, but per case, it was. Details are shown in Table 2. No difference was found in antimicrobial use between responders that defined case 1 as SEA and/or RAO (38% stated to use antimicrobials at least sometimes in case 1) vs. responders that did not use these terms (37%, *p* 0.81) nor between responders that did or did not use the terms Mild EA and/or IAD in case 2 (31% vs. 31%, *p* 0.94) or between responders that did or did not use the terms mild-to-moderate EA and/or moderate EA and/or IAD in case 3 (30% vs. 20%, *p* 0.13).

Concerning the use of diagnostic modalities, the proportion of veterinarians using antimicrobials was smaller amongst those who stated to use endoscopy, BAL, and radiology, and a similar trend was seen with participants using ultrasound and tracheal washes (Table 3). Antimicrobials were used by a similar proportion of veterinarians that did or did not use pleural pressure measurements and/or ancillary pulmonary function tests.

**Figure 5:**
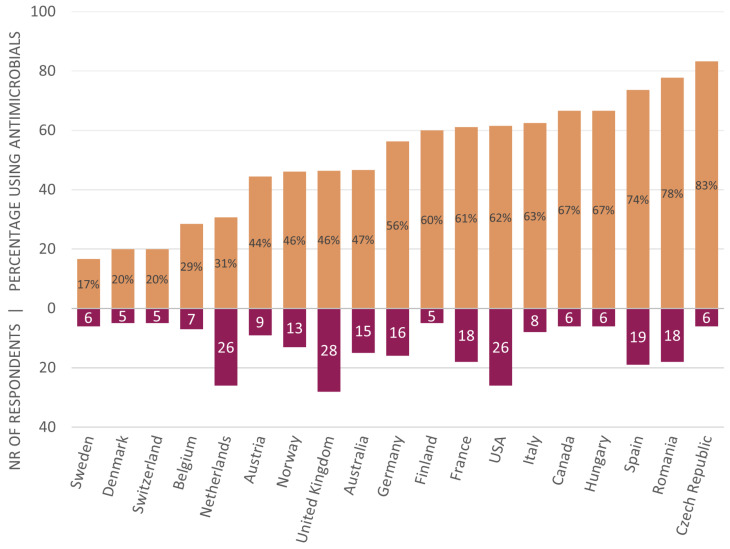
Antimicrobial use according to country. Countries with more than four respondents are shown.

The proportions of veterinarians stating to use antimicrobials differs per country (see Figure 5).

The most selected reason to use antimicrobials at least sometimes in all three cases was a suspicion of a secondary bacterial infection. The results are shown in Table 4.

##### Antimicrobial Type

**Figure 6:**
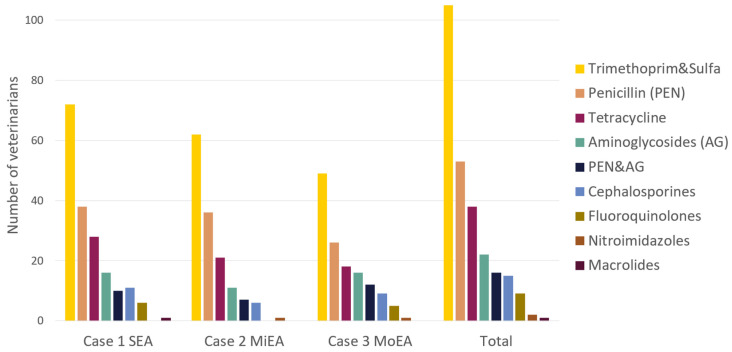
The types of antimicrobials used by the participants. The types used are similarly distributed throughout the cases.

Trimethoprim Sulfonamide combinations were most often prescribed by the participants in all three cases. In total, 80% of antimicrobial-using participants (105/131) selected TMS. Penicillins (40%) and tetracyclines (29%) were the second and third most selected antimicrobials, respectively (Figure 6).

The critically important antimicrobials, cephalosporins, fluoroquinolones, and macrolides, were selected by 15 (11%), 9 (7%), and 1 (<1%) of the respondents, respectively.

Of the 131 respondents using antimicrobials (at least sometimes in at least one of the cases), 105 reported the presence of national regulations in the country they worked in. Tetracyclines were used by more veterinarians (46%, 12/26) that stated no presence of national regulations compared to 25% (26/105) that did (*p* 0.031). Cephalosporins were also used more by veterinarians that reported no presence of national guidelines (23%, 6/26) compared to 9% (*n* = 9) of veterinarians that were aware of national regulations (*p* 0.038).

The same trend was visible between participants who did or did not state the presence of regulations in their practice, with a higher proportion (16%) using cephalosporins when no regulations were present compared to veterinarians with practice regulations (5%, *p* 0.058).

#### 3.3.2. Factors Influencing Prescription Behavior

##### Factors That Drive Prescription Behavior

The ten most selected factors were all medical (Table 5), while the seven least selected factors were all non-medical (Table 6). When asked why they did not select the factor ‘Whether the owner requests antimicrobials’, 15 out of 122 (12%) participants stated that it was because owners (almost) never request antimicrobials, and 102 (84%) stated that it was because owner requests did not influence their decision. The remaining 14 did not select an answer.

When asked why they did not select the factor ‘Whether it is general practice at the clinic/practice where you work to prescribe antimicrobials in a case like this’, 70 out of 123 (57%) participants stated that it was not general policy in their practice, and 48 (39%) stated that the ‘general practice’ of their colleagues did not influence their decision. The remaining 13 did not select an answer.

##### Direction of the Factors Driving Prescription Behavior

**Figure 7:**
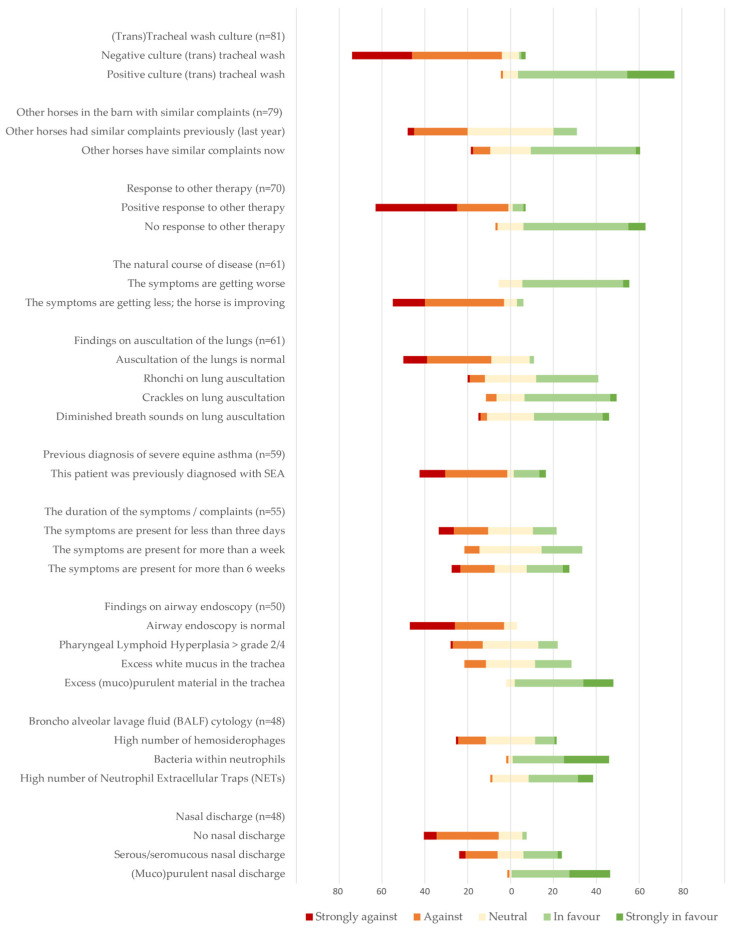
The proportion of respondents that consider that factor/patient information as ‘against’ or ‘in favor of’ prescribing antimicrobials. Ten most selected factors are shown.

Of the ten most selected factors as presented in Table 5, in Figure 7, it is shown how these factors influence the decisions of participants towards or away from prescribing antimicrobials.

For some factors, the direction is clear; most respondents consider a negative tracheal wash a factor against the prescription of antimicrobials and a positive tracheal wash a factor in favor of prescribing antimicrobials. However, when looking at the duration of symptoms or whether a horse has been diagnosed with severe equine asthma before, there is much less consensus. For example, of 55 participants who consider ‘duration of symptoms’ a factor of interest when considering antimicrobials, 20 (36%) state that when complaints exist for more than 6 weeks, this would be a reason to prescribe antimicrobials, but also 20 (36%) would consider that a reason against prescribing antimicrobials (see Figure 7).

## 4. Discussion

This survey-based study offers a comprehensive view of global veterinarians’ antimicrobial use and prescription behavior in equine asthma cases. Little has been published on this topic, although studies on prescription decisions and behavior in human health are available [15,24,25,26,27,34,35,36,37,38,39] as are fewer on antimicrobial prescriptions by veterinarians for their equine patients [16,21,28,30,40,41]. No other paper, though, has reported antimicrobial prescription behavior with a focus on asthmatic horses.

Of the 249 respondents that finished the survey, 53% stated to use antimicrobials at least sometimes in at least one of the cases, most in the severe equine asthma case (37%). This is in line with two previous reports on case-based surveys: Hughes et al. reported 53% of respondents prescribing antimicrobials in an adult coughing horse in first opinion practice in the United Kingdom [21], and Schwechler et al. (2016) reported 36% of Swiss, German, and Austrian practitioners prescribing antimicrobials in a case presented as recurrent airway obstruction [30]. In the American study by Rule et al. (2021), the prescription rate in respiratory cases is lower (24% of visits), but the type of respiratory disease was not further specified [41].

The respondents were practitioners and specialists with different career lengths, evenly represented. In people, although regarding diverse populations of patients, several studies have shown that career length can be a factor in antimicrobial prescription behavior. Akkerman et al. (2005) found that the frequency of antimicrobial prescriptions in patients presenting for upper respiratory tract infections (uRTIs) increased the longer the practitioner had practiced [25]. In Canada, a retrospective cohort study found the duration of antimicrobial therapy to be longer for late-career physicians [38]. In an Australian study, on the other hand, early career was associated with a lack of rational antimicrobial prescription for uRTIs and acute bronchitis cases [39]. In our study, career length was not a significant factor in the prescription rate in our equine asthma cases, similar to findings in other studies regarding antimicrobial prescription in horses [21,28,30].

The geographical distribution of the respondents was worldwide, with the majority working in Europe, the USA, Canada, and Australia (the unequal distribution will be discussed later). The antimicrobial use differed per country, with countries from western Europe using the least (17–20% of veterinarians in Sweden, Denmark, and Switzerland), via Australia (47%) and the USA (62%), to southern and eastern Europe prescribing the most (74–83% in Spain, Romania, and the Czech Republic). Reasons for this are likely diverse, for example, prescribing culture in general and the presence/awareness of national regulations. National regulations concerning antimicrobial use are increasingly being instituted worldwide to improve antimicrobial stewardship. Awareness of national regulations in their country was stated by 204 (82%) respondents from 24 (out of 25) countries. Interestingly, 27 of the 199 (13%) respondents from ten countries in Europe were not aware of national regulations, although all these countries are members of the European Surveillance of Veterinary Antimicrobial Consumption (ESVAC). In other studies, no report was made of awareness of national regulations, although the presence of practice guidelines was examined and found to be lower than the 46% of respondents in our study, with 13% by Schwechler et al. (2016) and 1% by Hughes et al. (2013) [21,30].

Awareness of national regulations does seem to be a factor in antimicrobial prescription, since 84% stated that they take these into account in every case in which they consider antimicrobials. Respondents were not directly asked whether they were aware of antimicrobial resistance development nor about their attitude towards (their role in) that, but they stated to consider these guidelines both as guidance (79%) and as a limitation (37%). This is in contrast, though, to the fact that we found no significant difference in the proportion of antimicrobial users in the group aware of national regulations compared to people who were not aware.

In people, a similar trend in geographical differences was seen in studies in five European countries with regard to the treatment of simulated asthma cases and a survey on knowledge concerning asthma and actual treatment choices in their patients [42,43]. Practitioners in The Netherlands prescribed fewer antimicrobials than practitioners in Norway and Sweden, while practitioners in Germany and the Slovak Republic prescribed the most antimicrobials.

An example of a cultural influence also seems present in Romania. The high prescription rate of antimicrobials among Romanian veterinarians mimics the same situation in human medicine; according to national statistics by the Romanian Health Ministry, 3% of the population uses antimicrobials daily [44], and according to ECDC, the country is first in the EU in regard to antimicrobial consumption [45]. The Czech Republic has a more responsible attitude towards antimicrobials in humans and is among the top non prescriber countries [45]. Interestingly, this was not consistent with the answers of the few respondents (n = 6) from the Czech Republic, as five out of six stated to prescribe antimicrobials in our cases. No data is available for Sweden on the ECDC surveillance list, but Denmark, which was a close second in the list with the lowest percentage of respondents using antimicrobials, has an appropriate prescription behavior [45]. In Sweden, there is strong knowledge on antimicrobial resistance and use, which might translate to the general public and veterinarians alike [46].

Also, in the study of Lagerløv, the knowledge on asthma exacerbations was higher among the practitioners from countries prescribing fewer antimicrobials [43], which is similar to our findings that people not using antimicrobials scored better on the knowledge questions. So not only awareness of regulations but also understanding of the pathophysiology of asthma seems related to less antimicrobial prescription, and dissemination of this knowledge may be an important factor in reducing antimicrobial use.

A complicating factor in interpreting our data is that using the definition of asthma is not necessarily the same as having knowledge of the pathophysiology. We did find that a smaller proportion of responders used antimicrobials when they used the term equine asthma to define the cases and also when they had more knowledge about the pathophysiologic background of equine asthma, but when including the terms from the previous consensus statement (IAD/RAO), no difference was found, even though these terms also relate to the pathophysiology.

A difference in knowledge may also in part explain the fact that more veterinarians working in first opinion practice (FOP) use antimicrobials in the presented cases, while people working in academic hospitals and private referral hospitals prescribe them less often.

Because of the heterogeneous character of asthma, optimal treatment probably differs between different populations regarding patient age, type of asthma, and severity (first opinion practice versus hospital-admitted patients), both in equine and in human medicine. This also complicates the (interpretation of) scientific research regarding the use and possible efficacy of antimicrobials in asthma cases.

In human medicine, studies are on hospitalized patients [9,15,37] or on patients in primary care [25,26,34,35,36], but no studies comparing these populations were found by the authors.

Bacterial infection is not considered a cause of equine asthma, although there is evidence for endotoxins as a trigger for asthma exacerbations [47]. Participants who assumed there was a role for bacteria in the pathophysiology of asthma did declare the use of antimicrobials often though, suggesting they considered infection likely despite the absence of fever. This is confirmed by the fact that over 75% (and even 82% in the severe equine asthma (SEA) case) stated a suspected secondary bacterial infection as a reason for prescribing antimicrobials. Interestingly, only 9% selected ‘quicker improvement’ and 10% selected ‘more improvement’ in their patients. The latter is in concordance with the little data there is on antimicrobials in equine asthma, where no clinical benefit was shown both as monotherapy and as a complementary treatment to conventional inhaled corticosteroid therapy [11,12]. While there is some evidence of bacterial presence in some particular cases of mild equine asthma in racehorses [48], there is no evidence that these are little more than commensals or concomitant lower respiratory tract infections [48,49]. Outcomes of antimicrobial addition to conventional treatment in horses with historical SEA that had a positive tracheal wash were investigated in a study in 2018, and the antimicrobial-treated group had improved clinical scores and myeloperoxidase activity, but other variables referring to inflammation were not affected at all [13].

Antimicrobial choice was consistent in all three cases; trimethoprim sulfonamide combinations were used most, followed by penicillin and tetracyclines. The use of TMS and penicillin is in line with the general use, registration, and availability of products for equine medicine. To the knowledge of the authors (EMA union database and FDA database [50,51]) there is no registered product (worldwide) for doxycyclin in horses, which was the product used by 76% of tetracycline prescribers. This may be the reason why it was used significantly more by people who were not aware of national regulations in the country they work in. Interestingly, in a paper on ambulatory prescription practices in the United States of America in 2021, the most used antimicrobials were aminoglycosides, with sulfonamides and tetracyclines in the second and third place, respectively [39], while in Europe antimicrobial use trends towards sulphonamide use, followed by penicillins and tetracyclines, much like our global results [21,40,52]. Of the critically important antimicrobials with the highest priority for human use as specified by the World Health Organization (WHO) [17] and considered critically important for veterinary use by the World Organization for Animal Health (WOAH, founded as OIE) [53], three were listed as options to select: cephalosporins, fluoroquinolones, and macrolides.

Cephalosporines were (together with the tetracyclines) used more by the group unaware of regulations. Although limited products with cephalosporins are registered and available, WOAH recommends that cephalosporins should not be used empirically or be the first-line choice of antimicrobial, and that ‘off-label’ use should be minimal.

Despite that, of the 131 veterinarians prescribing antimicrobials in these cases, 11% stated to choose cephalosporins sometimes. This was in line with cephalosporines being prescribed in 13% of visits in the American study from Rule et al. (2021) and the numbers found by Wilson et al. (2023), but more compared to the study of Hughes et al., where <2% used cephalosporines in the adult coughing horse [21,23,41]. In the study of Hughes et al., respondents working at referral practices were more likely to prescribe cephalosporins and fluoroquinolones [21]. This was not the case in our study. Fluoroquinolones, mostly enrofloxacin, were stated to be used by 7% of prescribers in our study, comparable again with the findings of Wilson et al. (2023), but more than in the studies of Rule (1.4%) and Hughes (<1%) [21,23,41]. Fluoroquinolones are, besides being on the highest priority list of the WHO, also considered protected by BEVA (the British Equine Veterinary Association) and EMA (the European Medicines Agency), restricting their use in animals in general (category B) [54].

In the past few years, a decrease in both cephalosporin and fluoroquinolone use has been noticed [55]. However, another recent survey by Wilson et al. (2023) on antimicrobial prescription in Europe and the UK reported respondents still using ceftiofur and enrofloxacin as a preference [23].

Macrolides were selected only once as a choice, and in the other studies, they do not seem to be used often in the adult horse.

Prescription behavior is, apart from awareness of (national) regulations or guidelines, prescription culture in the environment people work in, education on the pathophysiology of disease, and antimicrobial stewardship, also dependent on case-related medical and non-medical situation-related factors, both in human as well as in veterinary medicine [26,28,29].

In the study of Coenen et al. (2002) concerning the prescription behavior of general practitioners for coughing human patients, medical factors predominated. However, some non-medical factors were also influencing the participating practitioners, both patient-related, like ‘the patient needs quick recovery for work’ or ‘the patient asks for antimicrobials’ as well as prescriber-related, like ‘working under time pressure’, or ‘will be blamed for not having prescribed antimicrobials if it subsequently appears to be necessary’ [26]. In our survey, we see a similar and even more distinct pattern towards medical factors. All presented factors were selected at least once, with the top ten all being medical and the fewest selected seven factors all being non-medical, although one may consider the willingness or refusal of the owner to change the environment of the horse and the factor that the horse has to compete in two weeks (partly) medical. The fact that the majority of veterinarians is not changing their decision when owners ask for antimicrobials is consistent with a report on the complexity of combining ‘shared decision making’ with restrictive antimicrobial use [35]. However, it is not consistent with a human study that does report patient requests as a factor in the prescription of antimicrobials [26].

Several of the most selected factors guiding antimicrobial prescription, namely: a positive tracheal wash culture, other horses in the barn with similar complaints now, (muco)purulent nasal discharge, and (muco)purulent material in the trachea, seem to be consistent with suspicion of an infectious/bacterial component. That is also consistent with the fact that many respondents stated to consider a secondary bacterial infection a reason for prescribing antimicrobials, even though that does not fit the diagnosis of asthma as presented in this survey. Apart from pathophysiological knowledge, diagnosis insecurity has also been reported as a major factor in human prescription behavior [27].

This is compatible with our finding that people using the diagnostic modalities of endoscopy, BAL, and radiography prescribe antimicrobials less often. The use of antimicrobials was not significantly different between responders performing tracheal washes compared to responders who do not, although we do see that the people who do perform them consider the result to be an important discriminating factor in their decision.

As discussed before, a positive culture on a tracheal wash does not have to be clinically relevant in an asthma case, and several horses in a barn with asthma-like symptoms may reveal more about the management of the stable than infectious disease [31,56], especially in the absence of fever. However, in this study, there was no information given on the tracheal wash results of the cases presented, even though apparently this was an important factor for many veterinarians. If TW results had been provided (especially in a negative culture), this could have changed the outcome towards less antimicrobial use/users.

Changes in the peripheral blood are not expected in horses with Equine Asthma. The fact that 13 veterinarians actively reported using laboratory blood analysis on this type of patient, likely also reflects a form of diagnosis insecurity.

Limitations to this study were bias towards academia/specialists, the length of the survey, the paper cases, and the geographical distribution.

The proportion of specialists and people working in academia was higher among our respondents than in reality. However, many veterinarians from first-opinion (private) practices completed the survey, enough to make valuable comparisons.

Although the survey was long, a large number of respondents finished it, making this a very valuable survey. The length, however, might have tended to lead to uniform, identical answers by participants [57]. This study tried to mitigate this by using a comprehensive Likert scale. This type of Likert scale can also be subject to response bias [58], and the validity of the attitude measurement can be compromised due to social desirability as well as central tendency bias—the propensity to choose the neutral option, which depends on personality and culture [59]. It was tried to correct such biases with sufficient options as responses.

Participants were asked to answer the questions fairly, as to what they would actually do with this type of patient, and to answer without consulting external sources. However, this was not controlled.

The English survey was translated into five more languages. This was positive, as it gave more veterinarians the option to respond. A bias can be that some translation error possibilities might have occurred.

The authors are aware that presenting three paper cases is not the same as presenting a living patient; however, this was the most logical option to have in a survey. The fact that the people who had a higher score on the knowledge of the consensus statement also defined the cases more often as asthma suggests that the cases were representative and recognizable as asthma cases. Still, although the multidisciplinary expertise panel that compiled the equine asthma consensus statement of ACVIM/ECEIM came up with recommendations for case definition and the nomenclature of equine asthma, the paper poses multiple questions left unanswered. More data is needed, and the dissemination of medical knowledge might be an important driving force behind better prescription behavior due to an understanding of pathophysiology and clinical manifestation. An example of this was shown when prescription behavior regarding human asthma before and after an educational intervention was reported in a group of doctors in five European countries [60].

Finally, the geographical location of the respondents was not distributed equally over the world and was not correlated to the distribution of horses in the world. Europe, especially The Netherlands and the UK, and were overrepresented; North America and Australia were somewhat represented, but other parts of the world were not represented at all.

## 5. Conclusions

Because of the need for antimicrobial stewardship and the limited evidence for antimicrobial use in equine asthma, a reduction in antimicrobial use in equine asthma is essential. To accomplish this, there is a need to understand the prescription behavior of equine veterinarians. This survey gave insight into the prescription behavior, with 53% of the participants in this survey stating to use antimicrobials at least some times in at least one of the asthma cases presented. Up to 11% of these respondents selected critically important antimicrobials as their treatment of choice. Furthermore, it became clear that knowledge of the pathophysiology of asthma, the country/culture and the type of practice the veterinarian works in are all important (and likely partly related) factors influencing prescription behavior. On the patient level, medical factors clearly predominate, several of which seem to contribute to diagnosis uncertainty since they are factors that could also be compatible with infectious disease.

Hereby, we underline the need for further research on antimicrobials in equine asthma, the dissemination of knowledge of the pathophysiology of equine asthma, and clear (worldwide) guidelines on antimicrobial stewardship to diminish the inappropriate use of (critically important) antimicrobials in cases of equine asthma.

## Figures and Tables

**Table 1:** Case definition of case 1 (severe equine asthma case, SEA), 2 (mild equine asthma case MiEA), and 3 (moderate equine asthma case MoEA). Number of respondents are given (percentage of all respondents). Numbers in bold are the answers according to the revised consensus statement [31].

	Case 1 ‘SEA’	Case 2 ‘MiEA’	Case 3 ‘MoEA’
Bronchitis	36 (14%)	36 (14%)	30 (12%)
Chronic Obstructive Pulmonary Disease (COPD)	7 (3%)	2 (1%)	4 (2%)
Inflammatory Airway Disease (IAD)	45 (18%)	114 (46%)	91 (37%)
Recurrent Airway Obstruction (RAO)	62 (25%)	4 (2%)	19 (8%)
Mild Equine Asthma (MiEA)	12 (5%)	**96 (39%)**	57 (23%)
Mild to Moderate Equine Asthma (MMEA)	41 (16%)	52 (21%)	**105 (42%)**
Moderate Equine Asthma (MoEA)	59 (24%)	5 (2%)	**44 (18%)**
Severe Equine Asthma (SEA)	**94 (38%)**	0 (0%)	4 (2%)
**EQUINE ASTHMA**	**189 (76%)**	**144 (58%)**	**191 (77%)**
Other	9 (4%)	32 (13%)	9 (4%)
No answer	0 (0%)	3 (1%)	0 (0%)

**Table 2:** Proportions of veterinarians stated to use antimicrobials at least sometimes in at least one case, compared between groups based on several respondent characteristics. * Proportion of veterinarians stating that they use antimicrobials, at least sometimes in this case.

Respondent Characteristic			% Using Antimicrobials	*p*-Value (χ^2^)
Career length		0–10 years	58%	0.289
		11–20 years	47%	
		>20 years	53%	
National regulations stated		Yes (*n* = 204)	51%	0.443
		No (*n* = 45)	58%	
Diplomates ECEIM/ACVIM		Yes (*n* = 79)	44%	0.074
		No (*n* = 170)	56%	
Considering bacteria to have a causal role in EA		Yes (*n* = 63)	81%	<0.001
		No (*n* = 186)	43%	
Working in Academia		Yes (*n* = 71)	41%	0.019
		No (*n* = 178)	57%	
Working in FOP		Yes (*n* = 126)	63%	0.001
		No (*n* = 123)	42%	
Equine Veterinarians		>95% equine patients (*n* = 191)	47%	0.002
		<95% equine patients (*n* = 58)	71%	
Defining cases as equine asthma	CASE 1	Yes (*n* = 189)	30% *	<0.001
		No (*n* = 60)	62% *	
	CASE 2	Yes (*n* = 144)	26% *	0.037
		No (*n* = 105)	38% *	
	CASE 3	Yes (*n* = 191)	24% *	0.008
		No (*n* = 58)	41% *	
	ALL CASES	Yes (*n* = 226)	51%	0.087
		No (*n* = 23)	70%	

**Table 3:** Proportions of veterinarians stated to use antimicrobials at least sometimes in at least one case, compared between groups based on the use of several diagnostic modalities.

Diagnostic Modality		% Using Antimicrobials	*p*-Value (χ^2^)
Endoscopy	Yes (*n* = 206)	47%	<0.001
	No (*n* = 43)	79%	
Bronchoalveolar Lavage	Yes (*n* = 190)	45%	<0.001
	No (*n* = 59)	78%	
Ultrasound	Yes (*n* = 148)	48%	0.076
	No (*n* = 101)	59%	
Tracheal Wash	Yes (*n* = 136)	47%	0.054
	No (*n* = 113)	59%	
Radiology	Yes (*n* = 99)	35%	<0.001
	No (*n* = 150)	64%	

**Table 4:** Reasons participants state for their antimicrobial use in the presented cases.

	Case 1 (*n* = 93)	Case 2 (*n* = 77)	Case 3 (*n* = 69)
I suspect a primary bacterial component.	9	17	15
I suspect a secondary bacterial infection.	76	54	52
The horses improve more quickly.	7	7	7
The horses show more improvement.	8	8	9
Other	13	7	5

**Table 5:** The ten most commonly selected factors influencing veterinarians in their decision to (not) prescribe antimicrobials.

Rank	Most Selected Factors	% of Participants (*n* = 131)
1	(Trans) Tracheal wash culture	81 (62%)
2	Other horses in the barn with similar complaints	79 (60%)
3	Response to other forms of therapy (no AM)	70 (53%)
4	Natural course of the disease	61 (47%)
4	Auscultation	61 (47%)
6	Previous diagnosis of severe equine asthma	59 (45%)
7	Duration of symptoms/complaints	55 (42%)
8	Findings on Endoscopy	50 (38%)
9	BALF cell morphology	48 (37%)
9	Nasal discharge	48 (37%)

**Table 6:** The seven factors least selected influencing veterinarians in their decision to (not) prescribe antimicrobials.

Rank	Least Selected Factors	% of Participants (*n* = 131)
30	Whether the horse is insured for medical costs	1 (1%)
29	Time pressure	5 (4%)
26	Whether you think the owner would like you to prescribe antimicrobials	8 (6%)
26	Whether or not the owner is willing to change the horse’s environment	8 (6%)
26	Whether it is general practice at the clinic/practice where you work to prescribe antimicrobials in a case like this	8 (6%)
25	Owner requests antimicrobials	9 (7%)
24	The horse needs to compete (in an important competition) in 2 weeks’ time	15 (11%)

## Data Availability

The data that support the findings of this study are available from the corresponding author upon reasonable request and while maintaining confidentiality/anonymity of the respondents.

## Supplementary Materials

The following supporting information can be downloaded at: https://www.mdpi.com/article/10.3390/ani14030457/s1, File S1: The full survey.
