# Supplementary material for: Antimicrobial Prescription Behavior in Equine Asthma Cases: An International Survey
Source: Animals (Basel). 2024 Jan 30;14(3):457. doi: 10.3390/ani14030457 (PMC10854497; doi:10.3390/ani14030457)
Supplement: Supplementary file 1 [file animals-14-00457-s001.zip › animals-2817564-supplementary.pdf]

English

Part 1

Voor Nederlands; kies in het menu rechtsboven.

Per l'italiano, per favore seleziona la lingua nel menu in alto a destra.

Pentru limba română, vă rugăm accesați meniul din dreapta sus.

Para realizar la encuesta en español, selecciona la optión en el menu, arriba a la derecha.

Pour le français, veuillez le sélectionner dans le menu en haut à droite.

Welcome to this survey concerning antimicrobial use in Equine Asthma.

As you may not know: this survey is also suited to take on your mobile device; this is to make sure you can do it where and whenever it suits you best. You can pause and proceed at any time. However, once you started, your data will be submitted 2 weeks after your last activity.

This questionnaire consists of several parts; please take all of them without looking anything up (except maybe data of your practice); we would like to know what you actually think/feel/do on a daily basis.

All data will be analysed anonymously. It will at most be presented on country level.

We know it will ask some of your valuable time, so thank you so much for participating!

Here is a brief overview of what we are going to ask you, so you know what to expect.

Part 1 (3-5 min): information on you and your (type of) practice (to be able to relate this to the answers of the other parts).

Part 2 (4-6 min): questions concerning the current knowledge and terminology around Equine Asthma; we would like to know how wel the current knowledge and nomenclature has reached and / or is implicated by vets / veterinary specialists.

Part 3 (6-12 min):
a) Questions investigating how you interpret and approach three example cases (concerning antimicrobial use).
b) In part 3b we will investigate several factors that might influence your choices on (not) using antimicrobials.
- Which factors influence your decision?
- How (in what direction) do these factors influence your decision?

Part 4 (1 min): A few short questions concerning future research.

So, please get started and thank you!

Consent form

The researcher will maintain the confidentiality of the research data and all data will be used anonymously.
By submitting this form you are indicating that you have read the description of the study, and that you agree to the terms* as described.

**I have read the information letter for participants. I was able to ask additional questions. My questions have been answered sufficiently. I had enough time to decide whether I would like to participate. I know that participating in this study is completely voluntary. I have the rights to see how my data are stored.*
I give permission to use my data in concordance with the aims as described in the information letter. If there is reason to use the data for another research aim, I will be asked for my permission again.
I give permission to store my raw research data for 10 (ten) years after the end of this study (date publication of the results) for further analysis concerning this study (if applicable).

I agree to participate in this study.

- ◯ Yes
- ◯ No

Are you sure you do not want to participate? Please confirm. If so, thank you for your time.

If you do want to participate, please go back to the consent form (left arrow below).

- ◯ I do not want to participate (this will end the survey).

Part 1

Information about you and your practice

How long have you been practicing veterinary medicine?

- ◯ 0-10 years
- ◯ 11-20 years
- ◯ over 20 years

In which country do you practice (at this moment)?

- ◯ Australia
- ◯ Austria
- ◯ Belgium
- ◯ Canada
- ◯ Czech Republic
- ◯ Finland
- ◯ France
- ◯ Germany
- ◯ Hungary
- ◯ Ireland
- ◯ Italy
- ◯ Spain
- ◯ Netherlands
- ◯ New Zealand
- ◯ Norway
- ◯ Sweden
- ◯ United Kingdom
- ◯ [ ] United States of America (please specify state)
- ◯ [ ] Other, please specify

What gender you identify with?

- ◯ Male
- ◯ Female
- ◯ Other / prefer not to disclose

What percentage of the patients you see are equine (including donkeys)?

- ◯ <30%
- ◯ 30-70%
- ◯ 71-95%
- ◯ >95%

Of what species consists the remainder of your patients? (Multiple answers possible)

- ☐ Companian animals
- ☐ Farm animals

What type of practice are you in? (Multiple answers possible)

- ☐ First opinion practice
- ☐ Private referral clinic
- ☐ Academic (referral) clinic

How many veterinarians work in equine medicine in your practice?

- 1
- 2-5
- 6-10
- >10

What number of equine cases does your practice see per year?

- < 300 per year
- 300-2000 per year
- 2001-5000 per year
- >5000 per year

What number of equine cases do you see on average per week?

- <10 per week
- 10-25 per week
- 26-50 per week
- >50 per week

How many equine respiratory cases you estimate you see per week? Please LEAVE OUT horses with fever and/or with upper respiratory tract problems.

- 0-2 per week
- 3-5 per week

- ◯ 6-10 per week
- ◯ > 10 per week

Which of the following techniques do you use in the work-up of these equine respiratory cases? (Multiple answers possible)

- ☐ Endoscopy
- ☐ Broncho Alveolar Lavage (BAL)
- ☐ (trans)tracheal wash
- ☐ Pleural pressure measurement (esophageal balloon catheter technique)
- ☐ Additional Pulmonary function testing (flowmeter etc).
- ☐ Radiography
- ☐ Ultrasonography
- ☐ [ ] Other, namely

What is your educational background? (Multiple answers possible)

- ☐ I have a veterinary degree (DVM)
- ☐ I am a doctor of Philosophy (PhD)
- ☐ I have a national specialist title in Equine internal medicine
- ☐ I am an international internal medicine specialist (Dipl ECEIM or ACVIM)

In the country / state / province where I work we have local/national guidelines/regulations regarding use of (critical) antimicrobials in veterinary medicine.

- ◯ Yes
- ◯ No

How do these local/national guidelines/regulations influence you?

	Strongly disagree	Somewhat disagree	Neither agree nor disagree	Somewhat agree	Strongly agree
I consider them in every equine case in which I consider antimicrobials.	○	○	○	○	○
I feel guided by these guidelines/regulations.	○	○	○	○	○
I feel limited by these guidelines/regulations.	○	○	○	○	○

At our practice / clinic we have specific guidelines/regulations concerning use and choice of antimicrobials in equine cases.

- ○ Yes
- ○ No

How do these guidelines/regulations from your practice/clinic influence you?

	Strongly disagree	Somewhat disagree	Neither agree nor disagree	Somewhat agree	Strongly agree
I consider them in every equine case in which I consider antimicrobials.	○	○	○	○	○
I feel guided by these guidelines/regulations.	○	○	○	○	○

	Strongly disagree	Somewhat disagree	Neither agree nor disagree	Somewhat agree	Strongly agree
I feel limited by these guidelines/regulations.	○	○	○	○	○

Part 2

Part 2

Questions about Equine Asthma

In this part you will find ten questions regarding (the definition of) Equine Asthma as used in current literature, followed by a question regarding your opinion on the role some pathogens play in the pathophysiology of Equine Asthma. You are explicitly NOT supposed to look up information; please answer with your current knowledge / the way you use the definitions mentioned. The questions may be difficult; they are up to specialist-level; therefore there is also the 'I don't know option'.
Please remember that data will be processed anonymously and will only be used to assess how well current nomenclature has been integrated / accepted by specialists and practitioners all over the world, and if / how this might relate to antimicrobial use.

Mild to moderate equine asthma is a disease mainly characterised by...

- increased breathing effort at rest.
- occasional coughing and poor performance.
- frequent coughing
- airflow limitation based on esophageal balloon catheter technique.
- I don't know

Mild to moderate asthma is equivalent to...

- recurrent airway obstruction (RAO).
- chronic obstructive pulmonary disease (COPD).
- bronchitis.
- inflammatory airway disease (IAD).
- I don't know

Severe equine asthma is (most) equivalent to ...

- recurrent airway obstruction (RAO).
- chronic obstructive pulmonary disease (COPD).
- bronchitis.
- inflammatory airway disease (IAD).
- I don't know

Severe equine asthma is known to ...

- spontaneously improve without treatment.
- occur more frequently in horses older than 7 years.

- ◯ be associated with an increased percentage of bronchoalveolar lavage fluid (BALF) eosinophils.
- ◯ have no airflow limitation based on esophageal balloon catheter technique.
- ◯ I don't know

A genetic influence is suspected for ...

- ◯ mild to moderate equine asthma.
- ◯ severe equine asthma.
- ◯ neither mild to moderate nor severe equine asthma.
- ◯ both mild to moderate and severe equine asthma.
- ◯ I don't know

Airway hyperresponsiveness is a factor in ...

- ◯ mild to moderate equine asthma.
- ◯ severe equine asthma.
- ◯ neither mild to moderate nor severe equine asthma.
- ◯ both mild to moderate and severe equine asthma.
- ◯ I don't know

An increase in neutrophils (> 25% of all cells) in the BALF is a common finding in ...

- ◯ mild to moderate equine asthma.
- ◯ severe equine asthma.
- ◯ neither mild to moderate nor severe equine asthma.
- ◯ both mild to moderate and severe equine asthma.
- ◯ I don't know

Excess tracheobronchial mucus is a common finding in ...

- mild to moderate equine asthma.
- severe equine asthma.
- neither mild to moderate nor severe equine asthma.
- both mild to moderate and severe equine asthma.
- I don't know

Airway remodelling is a factor in ...

- mild to moderate equine asthma.
- severe equine asthma.
- neither mild to moderate nor severe equine asthma.
- both mild to moderate and severe equine asthma.
- I don't know

Mast cells and/or eosinophil increase in BALF is a common finding in ...

- mild to moderate equine asthma.
- severe equine asthma.
- neither mild to moderate nor severe equine asthma.
- both mild to moderate and severe equine asthma.
- I don't know

What is your opinion on the role of the following pathogens in the

pathophysiology of Equine Asthma?

	Strongly disagree	Somewhat disagree	Neither agree nor disagree	Somewhat agree	Strongly agree
I assume an important role for bacteria in some Equine Asthma cases.	○	○	○	○	○
I assume an important role for fungi in some Equine Asthma cases.	○	○	○	○	○
I assume an important role for virusses in some Equine Asthma cases.	○	○	○	○	○

Part 3a cases and antimicrobials

Part 3a

We will present you with three equine respiratory cases. Questions are on whether or not you would consider to use antimicrobials, and if so we'll ask you for details concerning why and which antimicrobials you use in these cases.

Case 1:
A 13yo Dutch Warmblood gelding, used for pleasure riding, is presented to you because he has been coughing for the last two weeks. Last year in the same period he had similar complaints that improved on your advices and treatment. He is housed at pasture during the day, stabled during the night. The horse is bright and alert, has a good body condition, a rectal temperature of 37.8°C (100°F), some nasal discharge (white mucus), and shows increased breathing effort (frequency 22/min, abdominal). He has a frequent productive cough. On auscultation you hear increased breath

sounds throughout the thorax and mild ronchi on the trachea and lungs. Airway endoscopy reveals marked amounts of mucus (grade 4/5). You consider to perform a BAL.

Do you treat this horse (or similar cases) with antimicrobials?

- ◯ Never
- ◯ Sometimes
- ◯ About half the time
- ◯ Most of the time
- ◯ Always

Case 1 (repeated):

A 13yo Dutch Warmblood gelding, used for pleasure riding, is presented to you because he has been coughing for the last two weeks. Last year in the same period he had similar complaints that improved on your advices and treatment. He is housed at pasture during the day, stabled during the night. The horse is bright and alert, has a good body condition, a rectal temperature of 37.8°C (100°F), some nasal discharge (white mucus), and shows increased breathing effort (frequency 22/min, abdominal). He has a frequent productive cough. On auscultation you hear increased breath sounds throughout the thorax and mild ronchi on the trachea and lungs. Airway endoscopy reveals marked amounts of mucus (grade 4/5). You consider to perform a BAL.

When I use antimicrobials in these cases this is because... (multiple answers possible, can be different in different cases)

- ☐ I suspect a primary bacterial component.
- ☐ I suspect a secondary bacterial infection.
- ☐ the horses improve more quickly.
- ☐ the horses show more improvement.
- ☐ [ ] Other, namely

Which antimicrobials do you use in cases like this? (multiple answers possible)
Note the ones you use orally, parenterally and/or per inhalation please.

- ☐ Trimethoprim - sulfa combinations
- ☐ Tetracyclines (e.g. oxytetracycline, doxycycline)
- ☐ Penicillins
- ☐ Aminoglycosides (e.g. gentamicin, kanamycin)
- ☐ Penicillin - Aminoglycoside combinations (please also tick the separate boxes in case you combine separate preparations)
- ☐ Cephalosporins (e.g. ceftiofur, cefquinome)
- ☐ Macrolides (e.g. azithromycin)
- ☐ Fluoroquinolones (e.g. enrofloxacin)
- ☐ Lincosamides (e.g. clindamycin)
- ☐ Nitroimidazoles (e.g. metronidazole)
- ☐ [ ] Other (including antifungals), namely

Case 1 (repeated):

A 13yo Dutch Warmblood gelding, used for pleasure riding, is presented to you because he has been coughing for the last two weeks. Last year in the same period he had similar complaints that improved on your advices and treatment. He is housed at pasture during the day, stabled during the night. The horse is bright and alert, has a good body condition, a rectal temperature of 37.8°C (100°F), some nasal discharge (white mucus), and shows increased breathing effort (frequency 22/min, abdominal). He has a frequent productive cough. On auscultation you hear increased breath sounds throughout the thorax and mild ronchi on the trachea and lungs. Airway endoscopy reveals marked amounts of mucus (grade 4/5).

The BAL result is: Macrophages 43%, Lymphocytes 29%, Neutrophils 27%, Mast cells 1%, Eosinophils 0%

How would you define a case like this? (multiple answers possible)

- ☐ Bronchitis
- ☐ Recurrent Airway Obstruction (RAO)
- ☐ Inflammatory Airway Disease (IAD)
- ☐ Chronic Obstructive Pulmonary disease (COPD)
- ☐ Mild Equine Asthma
- ☐ Mild to Moderate Equine Asthma
- ☐ Moderate Equine Asthma
- ☐ Severe Equine Asthma
- ☐ Other, namely

Case 2:

A 3yo thoroughbred mare is presented to you for recent suboptimal performance (racing). She occasionally coughs for a week now. A few other horses in the barn have been heard coughing as well. She is housed in a stable on wood shavings and eats good quality hay. She does not have a fever (37.6°C / 99.7°F). You do not find any orthopedic or cardiovascular abnormalities. On examination of the respiratory tract you find a normal breathing frequency (12/min), no dyspnea and no adventitious sounds on auscultation of the thorax and trachea. A little bit of seromucous nasal discharge is present in both nostrils. On endoscopy you see pharyngeal lymphoid hyperplasia (grade 3: pink and white follicles covering the pharyngeal walls and dorsal suface of the soft palate) and mild increase of mucus in the trachea (grade 2/5). You consider to perform a BAL.

Do you treat this horse (or similar cases) with antimicrobials?

- ○ Never

- ◯ Sometimes
- ◯ About half the time
- ◯ Most of the time
- ◯ Always

Case 2 (Repeated):

A 3yo thoroughbred mare is presented to you for recent suboptimal performance (racing). She occasionally coughs for a week now. A few other horses in the barn have been heard coughing as well. She is housed in a stable on wood shavings and eats good quality hay.

She does not have a fever (37.6°C / 99.7°F). You do not find any orthopedic or cardiovascular abnormalities. On examination of the respiratory tract you find a normal breathing frequency (12/min), no dyspnea and no adventitious sounds on auscultation of the thorax and trachea. A little bit of seromucous nasal discharge is present in both nostrils. On endoscopy you see pharyngeal lymphoid hyperplasia (grade 3: pink and white follicles covering the pharyngeal walls and dorsal suface of the soft palate) and mild increase of mucus in the trachea (grade 2/5). You consider to perform a BAL.

When I use antimicrobials in these cases this is because... (multiple answers possible, can be different in different cases)

- ☐ I suspect a primary bacterial component.
- ☐ I suspect a secondary bacterial infection.
- ☐ the horses improve more quickly.
- ☐ the horses show more improvement.
- ☐ [ ] Other, namely

Which antimicrobials do you use in cases like this? (multiple answers possible)

Note the ones you use orally, parenterally and/or per inhalation please.

- [ ] Trimethoprim - sulfa combinations
- [ ] Tetracyclines (e.g. oxytetracycline, doxycycline)
- [ ] Penicillins
- [ ] Aminoglycosides (e.g. gentamicin, kanamycin)
- [ ] Penicillin - Aminoglycoside combinations (please also tick the separate boxes in case you combine separate preparations)
- [ ] Cephalosporins (e.g. ceftiofur, cefquinome)
- [ ] Macrolides (e.g. azithromycin)
- [ ] Fluoroquinolones (e.g. enrofloxacin)
- [ ] Lincosamides (e.g. clindamycin)
- [ ] Nitroimidazoles (e.g. metronidazole)
- [ ] ______ Other (including antifungals), namely

Case 2 (Repeated):

A 3yo thoroughbred mare is presented to you for recent suboptimal performance (racing). She occasionally coughs for a week now. A few other horses in the barn have been heard coughing as well. She is housed in a stable on wood shavings and eats good quality hay.

She does not have a fever (37.6°C / 99.7°F). You do not find any orthopedic or cardiovascular abnormalities. On examination of the respiratory tract you find a normal breathing frequency (12/min), no dyspnea and no adventitious sounds on auscultation of the thorax and trachea. A little bit of seromucous nasal discharge is present in both nostrils. On endoscopy you see pharyngeal lymphoid hyperplasia (grade 3: pink and white follicles covering the pharyngeal walls and dorsal suface of the soft palate) and mild increase of mucus in the trachea (grade 2/5).

The BAL result is: Macrophages 57%, Lymphocytes 33%, Neutrophils 4%, Mast cells 2%, Eosinophils 4%
How would you define a case like this? (multiple answers possible)

- [ ] Bronchitis

- ☐ Recurrent Airway Obstruction (RAO)
- ☐ Inflammatory Airway Disease (IAD)
- ☐ Chronic Obstructive Pulmonary disease (COPD)
- ☐ Mild Equine Asthma
- ☐ Mild to Moderate Equine Asthma
- ☐ Moderate Equine Asthma
- ☐ Severe Equine Asthma
- ☐ [ ] Other, namely

Case 3:
A 9yo German Warmblood mare, a high level show-jumper, is presented to you because of poor performance. Especially at the last fences she seems to have more touches and faults. Also the owner noticed that it takes her longer to regain a normal breathing frequency after exercise.
Coughing has been heard mainly at the beginning of exercise. All complaints are present since approximately 2 weeks.
At rest, the general and respiratory clinical exams are unremarkable. On endoscopy you see increased mucus production (grade 3/5). You consider to perform a BAL.

Do you treat this horse (or similar cases) with antimicrobials?

- ○ Never
- ○ Sometimes
- ○ About half the time
- ○ Most of the time
- ○ Always

Case 3 (Repeated):

A 9yo German Warmblood mare, a high level show-jumper, is presented to you because of poor performance. Especially at the last fences she seems to have more touches and faults. Also the owner noticed that it takes her longer to regain a normal breathing frequency after exercise.

Coughing has been heard mainly at the beginning of exercise. All complaints are present since approximately 2 weeks.

At rest, the general and respiratory clinical exams are unremarkable. On endoscopy you see increased mucus production (grade 3/5). You consider to perform a BAL.

When I use antimicrobials in these cases this is because... (multiple answers possible, can be different in different cases)

- ☐ I suspect a primary bacterial component.
- ☐ I suspect a secondary bacterial infection.
- ☐ the horses improve more quickly.
- ☐ the horses show more improvement.
- ☐ [ ] Other, namely

Which antimicrobials do you use in cases like this? (multiple answers possible) Note the ones you use orally, parenterally and/or per inhalation please.

- ☐ Trimethoprim - sulfa combinations
- ☐ Tetracyclines (e.g. oxytetracycline, doxycycline)
- ☐ Penicillins
- ☐ Aminoglycosides (e.g. gentamicin, kanamycin)
- ☐ Penicillin - Aminoglycoside combinations (please also tick the separate boxes in case you combine separate preparations)
- ☐ Cephalosporins (e.g. ceftiofur, cefquinome)
- ☐ Macrolides (e.g. azithromycin)

- [ ] Fluoroquinolones (e.g. enrofloxacin)
- [ ] Lincosamides (e.g. clindamycin)
- [ ] Nitroimidazoles (e.g. metronidazole)
- [ ] Other (including antifungals), namely

Case 3 (Repeated):

A 9yo German Warmblood mare, a high level show-jumper, is presented to you because of poor performance. Especially at the last fences she seems to have more touches and faults. Also the owner noticed that it takes her longer to regain a normal breathing frequency after exercise.

Coughing has been heard mainly at the beginning of exercise. All complaints are present since approximately 2 weeks.

At rest, the general and respiratory clinical exams are unremarkable. On endoscopy you see increased mucus production (grade 3/5).

The BAL result is: Macrophages 49%, Lymphocytes 28%, Neutrophils 16%, Mast cells 6%, Eosinophils 1%
How would you define a case like this? (multiple answers possible)

- [ ] Bronchitis
- [ ] Recurrent Airway Obstruction (RAO)
- [ ] Inflammatory Airway Disease (IAD)
- [ ] Chronic Obstructive Pulmonary disease (COPD)
- [ ] Mild Equine Asthma
- [ ] Mild to Moderate Equine Asthma
- [ ] Moderate Equine Asthma
- [ ] Severe Equine Asthma
- [ ] Other, namely

You stated to use trimethoprim-sulfa combinations in (at least) one of the case-examples.
By which route do you administer/prescribe trimethoprim-sulfa combinations when used in these cases? (multiple answers possible)

- [ ] Per inhalation
- [ ] Oral
- [ ] Intravenous
- [ ] Intramuscular

You stated to use tetracyclines in (at least) one of the case-examples.
Which tetracyclines do you use in these cases? (multiple answers possible).

- [ ] (Oxy)tetracyline
- [ ] Doxycycline
- [ ] Chlortetracycline
- [ ] ________ Other, namely

By which route do you administer (oxy)tetracycline when used in these cases? (multiple answers possible)

- [ ] Per inhalation
- [ ] Oral
- [ ] Intravenous
- [ ] Intramuscular

By which route do you administer/prescribe doxycycline when used in these cases? (multiple answers possible)

- ☐ Per inhalation
- ☐ Oral
- ☐ Intravenous
- ☐ Intramuscular

By which route do you administer/prescribe chlortetracycline when used in these cases? (multiple answers possible)

- ☐ Per inhalation
- ☐ Oral
- ☐ Intravenous
- ☐ Intramuscular

By which route do you administer/prescribe ${q://QID43/ChoiceTextEntryValue/3} when used in these cases? (multiple answers possible)

- ☐ Per inhalation
- ☐ Oral
- ☐ Intravenous
- ☐ Intramuscular

You stated to use penicillin - aminoglycoside-combination(s) in (at least) one of the case-examples.
Which penicillin - aminoglycoside-combination preparations do you use in these cases? (multiple answers possible).

- ☐ Procaine benzylpenicillin and (dihydro)streptomycin
- ☐ Procaine benzylpenicillin and neomycin
- ☐ [ ] Other, namely
- ☐ I combine separate preparations (to be specified in the next questions)

By which route do you administer/prescribe the procaine benzylpenicillin and dihydrostreptomycin-combination when used in these cases? (multiple answers possible)

- ☐ Per inhalation
- ☐ Oral
- ☐ Intravenous
- ☐ Intramuscular

By which route do you administer/prescribe the procaine benzylpenicillin and neomycin-combination when used in these cases? (multiple answers possible)

- ☐ Per inhalation
- ☐ Oral
- ☐ Intravenous
- ☐ Intramuscular

By which route do you administer/prescribe ${q://QID237/ChoiceTextEntryValue/4} when used in these cases? (multiple answers possible)

- ☐ Per inhalation
- ☐ Oral
- ☐ Intravenous
- ☐ Intramuscular

You stated to use penicillins in (at least) one of the case-examples. Which penicillins do you use in these cases? (multiple answers possible).

- ☐ Benzyl penicillin (-sodium)
- ☐ Procaine benzylpenicillin
- ☐ Amoxicillin
- ☐ Ampicillin
- ☐ Bacampicillin
- ☐ [ ] Other, namely

By which route do you administer/prescribe benzyl penicillin (-sodium) when used in these cases? (multiple answers possible)

- ☐ Per inhalation
- ☐ Oral
- ☐ Intravenous
- ☐ Intramuscular

By which route do you administer/prescribe procaine benzyl penicillin when used in these cases? (multiple answers possible)

- [ ] Per inhalation
- [ ] Oral
- [ ] Intravenous
- [ ] Intramuscular

By which route do you administer/prescribe amoxicillin when used in these cases? (multiple answers possible)

- [ ] Per inhalation
- [ ] Oral
- [ ] Intravenous
- [ ] Intramuscular

By which route do you administer/prescribe ampicillin when used in these cases? (multiple answers possible)

- [ ] Per inhalation
- [ ] Oral
- [ ] Intravenous
- [ ] Intramuscular

By which route do you administer/prescribe bacampicillin when used in these cases? (multiple answers possible)

- [ ] Per inhalation
- [ ] Oral

- [ ] Intravenous
- [ ] Intramuscular

By which route do you administer/prescribe ${q://QID44/ChoiceTextEntryValue/4} when used in these cases? (multiple answers possible)

- [ ] Per inhalation
- [ ] Oral
- [ ] Intravenous
- [ ] Intramuscular

You stated to use aminoglycosides in (at least) one of the case-examples.
Which aminoglycosides do you use in these cases? (multiple answers possible).

- [ ] Gentamicin
- [ ] Kanamycin
- [ ] Streptomycin
- [ ] Neomycin
- [ ] Amikacin
- [ ] ________ Other, namely

By which route do you administer/prescribe gentamicin when used in these cases? (multiple answers possible)

- [ ] Per inhalation

- [ ] Oral
- [ ] Intravenous
- [ ] Intramuscular

By which route do you administer/prescribe kanamycin when used in these cases? (multiple answers possible)

- [ ] Per inhalation
- [ ] Oral
- [ ] Intravenous
- [ ] Intramuscular

By which route do you administer/prescribe streptomycin when used in these cases? (multiple answers possible)

- [ ] Per inhalation
- [ ] Oral
- [ ] Intravenous
- [ ] Intramuscular

By which route do you administer/prescribe neomycin when used in these cases? (multiple answers possible)

- [ ] Per inhalation
- [ ] Oral
- [ ] Intravenous
- [ ] Intramuscular

By which route do you administer/prescribe amikacin when used in these cases? (multiple answers possible)

- ☐ Per inhalation
- ☐ Oral
- ☐ Intravenous
- ☐ Intramuscular

By which route do you administer/prescribe ${q://QID45/ChoiceTextEntryValue/4} when used in these cases? (multiple answers possible)

- ☐ Per inhalation
- ☐ Oral
- ☐ Intravenous
- ☐ Intramuscular

You stated to use cephalosporins in (at least) one of the case-examples.
Which cephalosporins do you use in these cases? (multiple answers possible).

- ☐ Ceftiofur
- ☐ Cefquinome
- ☐ [ ] Other, namely

By which route do you administer/prescribe ceftiofur when used in these cases? (multiple answers possible)

- [ ] Per inhalation
- [ ] Oral
- [ ] Intravenous
- [ ] Intramuscular

By which route do you administer/prescribe cefquinome when used in these cases? (multiple answers possible)

- [ ] Per inhalation
- [ ] Oral
- [ ] Intravenous
- [ ] Intramuscular

By which route do you administer/prescribe ${q://QID46/ChoiceTextEntryValue/3} when used in these cases? (multiple answers possible)

- [ ] Per inhalation
- [ ] Oral
- [ ] Intravenous
- [ ] Intramuscular

You stated to use macrolides in (at least) one of the case-examples. Which macrolides do you use in these cases? (multiple answers possible).

- ☐ Azithromycin
- ☐ Erythromycin
- ☐ Clarithromycin
- ☐ [ ] Other, namely

By which route do you administer/prescribe azithromycin when used in these cases? (multiple answers possible)

- ☐ Per inhalation
- ☐ Oral
- ☐ Intravenous
- ☐ Intramuscular

By which route do you administer/prescribe erythromycin when used in these cases? (multiple answers possible)

- ☐ Per inhalation
- ☐ Oral
- ☐ Intravenous
- ☐ Intramuscular

By which route do you administer/prescribe clarithromycin when used in these cases? (multiple answers possible)

- ☐ Per inhalation
- ☐ Oral
- ☐ Intravenous
- ☐ Intramuscular

By which route do you administer/prescribe ${q://QID47/ChoiceTextEntryValue/4} when used in these cases? (multiple answers possible)

- ☐ Per inhalation
- ☐ Oral
- ☐ Intravenous
- ☐ Intramuscular

You stated to use fluoroquinolones in (at least) one of the case-examples.
Which fluoroquinolones do you use in these cases? (multiple answers possible).

- ☐ Enrofloxacin
- ☐ Marbofloxacin
- ☐ [ ] Other, namely

By which route do you administer/prescribe enrofloxacin when used in these cases? (multiple answers possible)

- ☐ Per inhalation
- ☐ Oral
- ☐ Intravenous
- ☐ Intramuscular

By which route do you administer/prescribe marbofloxacin when used in these cases? (multiple answers possible)

- ☐ Per inhalation
- ☐ Oral
- ☐ Intravenous
- ☐ Intramuscular

By which route do you administer/prescribe ${q://QID48/ChoiceTextEntryValue/3} when used in these cases? (multiple answers possible)

- ☐ Per inhalation
- ☐ Oral
- ☐ Intravenous
- ☐ Intramuscular

You stated to use lincosamides in (at least) one of the case-examples.
Which lincosamides do you use in these cases? (multiple answers possible).

- ☐ Clindamycin
- ☐ [ ] Other, namely

By which route do you administer/prescribe clindamycin when used in these cases? (multiple answers possible)

- [ ] Per inhalation
- [ ] Oral
- [ ] Intravenous
- [ ] Intramuscular

By which route do you administer/prescribe ${q://QID230/ChoiceTextEntryValue/2} when used in these cases? (multiple answers possible)

- [ ] Per inhalation
- [ ] Oral
- [ ] Intravenous
- [ ] Intramuscular

You stated to use nitroimidazoles in (at least) one of the case-examples.
Which nitroimidazole do you use in these cases? (multiple answers possible).

- [ ] Metronidazole
- [ ] ______ Other, namely

By which route do you administer/prescribe metronidazole when used in these cases? (multiple answers possible)

- [ ] Per inhalation
- [ ] Oral
- [ ] Intravenous

- [ ] Intramuscular

By which route do you administer/prescribe ${q://QID234/ChoiceTextEntryValue/2} when used in these cases? (multiple answers possible)

- [ ] Per inhalation
- [ ] Oral
- [ ] Intravenous
- [ ] Intramuscular

You stated to use ${q://QID49/ChoiceTextEntryValue/8} in case-example 1.
By which route do you administer/prescribe ${q://QID49/ChoiceTextEntryValue/8} when used in these cases? (multiple answers possible)

- [ ] Per inhalation
- [ ] Oral
- [ ] Intravenous
- [ ] Intramuscular

You stated to use ${q://QID41/ChoiceTextEntryValue/8} in case-example 2.
By which route do you administer/prescribe ${q://QID41/ChoiceTextEntryValue/8} when used in these cases? (multiple answers possible)

- [ ] Per inhalation

- [ ] Oral
- [ ] Intravenous
- [ ] Intramuscular

You stated to use ${q://QID258/ChoiceTextEntryValue/8} in case-example 3.
By which route do you administer/prescribe ${q://QID258/ChoiceTextEntryValue/8} when used in these cases? (multiple answers possible)

- [ ] Per inhalation
- [ ] Oral
- [ ] Intravenous
- [ ] Intramuscular

You checked the box for using inhalation as a route for antimicrobial administration. What type of device do you use to do this? Multiple answers are possible. If you wish to clearify a few details (e.g. when using different techniques for different antimicrobials), please do so in the text box below.

- [ ] Jet nebulizer
- [ ] Ultrasonic nebulizer
- [ ] Mist inhaler

The device I use is aiming at

- [ ] local administration (e.g. facemask over the nose as in the Flexineb® system)
- [ ] 'room' administration (e.g. special closed box where the horse is placed for a while)

If you would like to share more details considering your experience with inhalation of antimicrobials; please do so here:

Part 3b factors

Because you never use antimicrobials in these cases, you will skip part 3b and now proceed to part 4!

Part 3b

In this part we will ask about factors affecting prescription behaviour.
First we will ask you *which* factors play a role in your decision to prescribe or not to prescribe antimicrobials. For factors that you do consider, we will ask how strongly and in what direction these factors influence your decision: against or in favour of antimicrobial prescription?

To make it clear, we will first look at an example:

EXAMPLE

If you select a factor that means you (at least sometimes) consider a certain factor, for example "peripheral blood

leukocyte count".
Then the follow-up question will be about how that factor plays a role in your decision to prescribe or not to prescribe antimicrobials. Does that factor *support* the prescription of antimicrobials or would it *argue against* prescribing antimicrobials?

In the example of the factor "peripheral blood leukocyte count": if you would like to point out that a leukopenia would strongly support antimicrobial use, while leukocytosis would (less strongly) support you in prescribing antimicrobials, this is how you would answer the question:

Do these factors make you decide against or in favour of the prescription of antimicrobials?

	Strongly Against	Against	Neutral	In favour	Strongly in favour
Leukopenia (<3 $x10^3$/μL)	○	○	○	○	◉
Leukocytosis (>12 $x10^3$/μL)	○	○	○	◉	○

We will now proceed with the real questions...

Below several factors are stated that may influence your decision to prescribe (or not to prescribe) antimicrobials.

Consider for these questions a patient (> 1 year old) with respiratory complaints (at rest or during exercise) that you suspect to have equine asthma.

To make sure we are considering the same patients: we define asthma as a clinical syndrome of respiratory complaints of variable severity that are caused by an inflammatory respons in the lower airways. Infectious pneumonia and/or pleuritis or other respiratory disease causing fever and general illness are EXCLUDED and not considered in these questions.

It is quite an extended list, please take a little time to really think about the factors and select which factors help you to decide for or against antimicrobial prescription.

- [ ] Whether this patient was previously diagnosed with severe equine asthma.
- [ ] Whether this is the first consult for this problem in this horse.
- [ ] Whether the horse does responds to other forms of therapy (corticosteroids and environmental change).
- [ ] Whether the horse responded to antimicrobial therapy before.
- [ ] The environment the horse is living in.
- [ ] Whether other horses in the barn have (had) similar complaints.
- [ ] Whether the horse shows exercise intolerance.
- [ ] Whether the horse is losing weight.
- [ ] Whether and how frequently the horse coughs.
- [ ] The type (quality) of cough.
- [ ] The duration of the symptoms / complaints.
- [ ] The natural course of the disease (symptoms getting worse or improving).
- [ ] Your general impression of the horse (e.g. body condition score (BCS) and hair coat)
- [ ] Whether the horse has nasal discharge.
- [ ] The respiratory rate and breathing pattern of the horse.
- [ ] Findings during auscultation of the lungs.
- [ ] Findings during airway endoscopy (e.g. excess tracheal mucus, pharyngeal hyperplasia, etc.).
- [ ] Broncho alveolar lavage fluid (BALF) cell distribution.

- ☐ Broncho alveolar lavage fluid (BALF) cytology (cell morphology, hemosiderophagia, etc.)
- ☐ BALF culture
- ☐ (Trans)Tracheal wash culture
- ☐ Radiographs of the thorax
- ☐ Ultrasonography of the thorax
- ☐ Whether or not the horse is insured for medical costs.
- ☐ Whether you think the owner would like you to prescribe antimicrobials.
- ☐ Whether or not the owner is willing to change the horse’s environment.
- ☐ Whether or not the horse needs to compete (in an important competition) in 2 weeks time.
- ☐ Whether you work under time pressure.
- ☐ Whether the owner requests for antimicrobials.
- ☐ Whether it is general practice at the clinic/practice where you work to prescribe antimicrobials in a case like this.

Do these factors make you decide against or in favour of the prescription of antimicrobials?

	Strongly against	Against	Neutral	In favour	Strongly in favour
This patient was previously diagnosed with severe equine asthma.	○	○	○	○	○

	Strongly against	Against	Neutral	In favour	Strongly in favour
This is the first consult for this problem in this horse.	○	○	○	○	○

	Strongly against	Against	Neutral	In favour	Strongly in favour
The horse DOES respond to other forms of therapy (inhaled corticosteroids and environmental change).	○	○	○	○	○
The horse does NOT respond to other forms of therapy (inhaled corticosteroids and environmental change).	○	○	○	○	○

	Strongly against	Against	Neutral	In favour	Strongly in favour
The horse responded to antimicrobial therapy before.	○	○	○	○	○
The horse did not respond to antimicrobial therapy before.	○	○	○	○	○

Do these factors make you decide against or in favour of the prescription of antimicrobials?

	Strongly against	Against	Neutral	In favour	Strongly in favour
The environment of the horse is well managed.	○	○	○	○	○
The environment of the horse is dusty.	○	○	○	○	○

	Strongly against	Against	Neutral	In favour	Strongly in favour
Other horses in the barn have similar complaints now	○	○	○	○	○
Other horses in the barn had similar complaints previously (last year)	○	○	○	○	○

Do these factors make you decide against or in favour of the prescription of antimicrobials?

	Strongly against	Against	Neutral	In favour	Strongly in favour
The horse shows exercise intolerance.	○	○	○	○	○

	Strongly against	Against	Neutral	In favour	Strongly in favour
The horse is losing weight.	○	○	○	○	○

	Strongly against	Against	Neutral	In favour	Strongly in favour
The horse does not cough.	○	○	○	○	○
The horse coughs sporadically (few times daily or only at the start of exercise).	○	○	○	○	○
The horse coughs frequently.	○	○	○	○	○

	Strongly against	Against	Neutral	In favour	Strongly in favour
The cough seems productive.	○	○	○	○	○

	Strongly against	Against	Neutral	In favour	Strongly in favour
The cough seems dry.	○	○	○	○	○

Do these factors make you decide against or in favour of the prescription of antimicrobials?

	Strongly Against	Against	Neutral	In favour	Strongly in favour
The symptoms are present for less than three days.	○	○	○	○	○
The symptoms are present for more than a week.	○	○	○	○	○
The symptoms are present for more than 6 weeks.	○	○	○	○	○

	Strongly Against	Against	Neutral	In favour	Strongly in favour
The symptoms are getting worse.	○	○	○	○	○
The symptoms are getting less; the horse is improving.	○	○	○	○	○

Do these factors make you decide against or in favour of the prescription of antimicrobials?

	Strongly against	Against	Neutral	In favour	Strongly in favour
Your general impression is not good: e.g the horse is dull, has a rough hair coat and has a low Body Condition Score.	○	○	○	○	○
Your general impression of the horse is good; the horse is bright and alert, has a shiny coat and a good Body Condition Score.	○	○	○	○	○

	Strongly Against	Against	Neutral	In favour	Strongly in favour
The horse has no nasal discharge.	○	○	○	○	○
The horse has serous/seromucous nasal discharge.	○	○	○	○	○
The horse has (muco)purulent nasal discharge.	○	○	○	○	○

Do these factors make you decide against or in favour of the prescription of antimicrobials?

	Strongly Against	Against	Neutral	In favour	Strongly in favour
The horse has a normal respiratory rate.	○	○	○	○	○
The horse has an increased respiratory rate.	○	○	○	○	○
The horse shows increased respiratory effort at rest.	○	○	○	○	○
The horse shows dyspnoea.	○	○	○	○	○

	Strongly Against	Against	Neutral	In favour	Strongly in favour
Auscultation of the lungs is normal.	○	○	○	○	○
On auscultation of the lungs ronchi are present.	○	○	○	○	○
On auscultation of the lungs crackles are present.	○	○	○	○	○
On auscultation of the lungs breath sounds are diminished.	○	○	○	○	○

Do these factors make you decide against or in favour of the prescription of antimicrobials?

	Strongly Against	Against	Neutral	In favour	Strongly in favour
Airway endoscopy is normal.	○	○	○	○	○
Pharyngeal Lymphoid Hyperplasia (Follicular pharyngitis) > grade 2/4	○	○	○	○	○
Excess white mucus is present in the trachea.	○	○	○	○	○
Excess (muco)purulent material is present in the trachea.	○	○	○	○	○

Do these factors make you decide against or in favour of the prescription of antimicrobials?

	Strongly Against	Against	Neutral	In favour	Strongly in favour
BALF neutrophil count > 25%	○	○	○	○	○
BALF neutrophil count >10% (-24%)	○	○	○	○	○
BALF mast cell count > 5%	○	○	○	○	○
BALF eosinophil count > 5%	○	○	○	○	○

	Strongly Against	Against	Neutral	In favour	Strongly in favour
High number of hemosiderophages	○	○	○	○	○
Bacteria within neutrophils	○	○	○	○	○
High number of Neutrophil Extracellular Traps (NETs)	○	○	○	○	○

	Strongly Against	Against	Neutral	In favour	Strongly in favour
Positive culture BALF	○	○	○	○	○

	Strongly Against	Against	Neutral	In favour	Strongly in favour
Negative culture (trans)tracheal wash	○	○	○	○	○
Positive culture (trans)tracheal wash	○	○	○	○	○

Do these factors make you decide against or in favour of the prescription of antimicrobials?

	Strongly Against	Against	Neutral	In favour	Strongly in favour
No abnormalities are presents on thoracic radiographs.	◯	◯	◯	◯	◯
On radiographs you see a diffuse bronchial pattern	◯	◯	◯	◯	◯
On radiographs you see a diffuse interstitial lung pattern.	◯	◯	◯	◯	◯

	Strongly Against	Against	Neutral	In favour	Strongly in favour
On ultrasound you see no abnormalities	◯	◯	◯	◯	◯
On ultrasound you see increased comet-tail artifacts.	◯	◯	◯	◯	◯

Do these factors make you decide against or in favour of the prescription of antimicrobials?

	Strongly Against	Against	Neutral	In favour	Strongly in favour
The horse is insured for medical costs.	○	○	○	○	○
The horse is NOT insured for medical costs.	○	○	○	○	○

	Strongly Against	Against	Neutral	In favour	Strongly in favour
You think the owner would like you to prescribe antimicrobials.	○	○	○	○	○

	Strongly Against	Against	Neutral	In favour	Strongly in favour
The owner is not willing to change the horse's environment.	○	○	○	○	○

	Strongly Against	Against	Neutral	In favour	Strongly in favour
The horse needs to compete (in an important competition) in 2 weeks time.	○	○	○	○	○

	Strongly Against	Against	Neutral	In favour	Strongly in favour
You work under time pressure.	○	○	○	○	○

Does this factor make you decide against or in favour of the prescription of antimicrobials?

	Strongly Against	Against	Neutral	In favour	Strongly in favour
The owner requests for antimicrobials.	○	○	○	○	○

You did not select 'owners asking for antimicrobials' as a factor to influence your decision on antimicrobial prescription.

Why not? Because...

- ○ owners (almost) never request for antimicrobials.
- ○ when owners do request for antimicrobials that does not influence my decision.

Does this factor make you decide against or in favour of the prescription of antimicrobials?

	Strongly Against	Against	Neutral	In favour	Strongly in favour
It is general practice at the clinic/practice where you work to prescribe antimicrobials in a case like this.	◯	◯	◯	◯	◯

You did not select 'the general practice concerning antimicrobial use in asthma patients in your clinic' as a factor to influence your decision on antimicrobial prescription.

Why not? Because...

- ◯ it is not general policy in the practice I work in.
- ◯ the 'general practice' of my colleagues does not influence my decision.

Part 4

Part 4

In this very short final part we would like to ask you about future research.

In case we would start a multicenter clinical trial concerning antimicrobial use in equine asthma, as a sequel to this questionnaire, would you be interested in participating?

If you are okay with us contacting you for this, please leave your e-mail address below.

- No thank you
- Maybe / probably / yes; my e-mail address:

Maybe we can collaborate?!
Do you have access to interesting techniques (e.g. determining biomarkers or Immunologic typing in BALF) and would you be willing to participate in future research using these?
If so, please leave your e-mail address.

- No
- Yes (or maybe); my e-mail address:

Would you like to be informed about the results of this questionnaire? If so, please leave your e-mail address.

- No thank you
- Yes; my e-mail address:

We are now at the end!

Thank you so much for completing this questionnaire!

Powered by Qualtrics
